# Machine learning implicates the IL-18 signaling axis in severe asthma

**DOI:** 10.1172/jci.insight.149945

**Published:** 2021-11-08

**Authors:** Matthew J. Camiolo, Xiuxia Zhou, Qi Wei, Humberto E. Trejo Bittar, Naftali Kaminski, Anuradha Ray, Sally E. Wenzel

**Affiliations:** 1 Division of Pulmonary, Allergy, and Critical Care Medicine, Department of Medicine, University of Pittsburgh School of Medicine, Pittsburgh, Pennsylvania, USA.; 2 Center for Systems Immunology, University of Pittsburgh, Pittsburgh, Pennsylvania, USA.; 3 Department of Environmental and Occupational Health, Graduate School of Public Health, University of Pittsburgh School of Medicine, Pittsburgh, Pennsylvania, USA.; 4 Department of Pathology, University of Pittsburgh Medical Center, Pittsburgh, Pennsylvania, USA.; 5 Pulmonary, Critical Care and Sleep Medicine, Yale School of Medicine, New Haven, Connecticut, USA.; 6 Department of Immunology, University of Pittsburgh School of Medicine, Pittsburgh, Pennsylvania, USA.

**Keywords:** Immunology, Pulmonology, Asthma, Bioinformatics, Molecular pathology

## Abstract

Asthma is a common disease with profoundly variable natural history and patient morbidity. Heterogeneity has long been appreciated, and much work has focused on identifying subgroups of patients with similar pathobiological underpinnings. Previous studies of the Severe Asthma Research Program (SARP) cohort linked gene expression changes to specific clinical and physiologic characteristics. While invaluable for hypothesis generation, these data include extensive candidate gene lists that complicate target identification and validation. In this analysis, we performed unsupervised clustering of the SARP cohort using bronchial epithelial cell gene expression data, identifying a transcriptional signature for participants suffering exacerbation-prone asthma with impaired lung function. Clinically, participants in this asthma cluster exhibited a mixed inflammatory process and bore transcriptional hallmarks of NF-**κ**B and activator protein 1 (AP-1) activation, despite high corticosteroid exposure. Using supervised machine learning, we found a set of 31 genes that classified patients with high accuracy and could reconstitute clinical and transcriptional hallmarks of our patient clustering in an external cohort. Of these genes, *IL18R1* (IL-18 Receptor 1) negatively associated with lung function and was highly expressed in the most severe patient cluster. We validated *IL18R1* protein expression in lung tissue and identified downstream NF-**κ**B and AP-1 activity, supporting IL-18 signaling in severe asthma pathogenesis and highlighting this approach for gene and pathway discovery.

## Graphical abstract

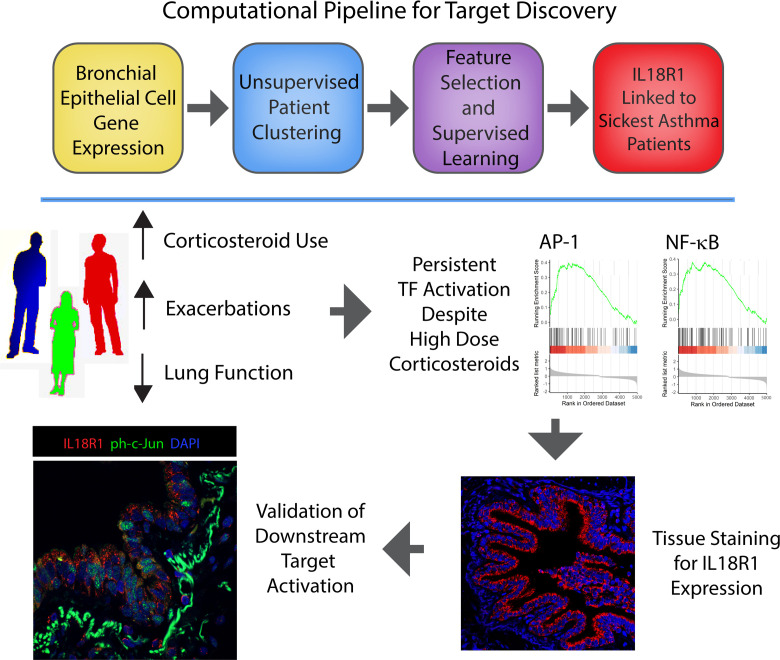

## Introduction

The understanding that asthma is a heterogeneous disease has long been appreciated (1). In the age of molecular targeted therapies, this led to the pursuit of treatments tailored to precise pathobiological mechanisms (2). We now understand that, depending on disease severity, 40%–70% of patients with asthma exhibit heightened Type-2 (T2) inflammation, which clinically improves with IL-4– and IL-5–targeted therapies (3, 4). Response to these therapeutics is not uniform, however, even in patients with biomarker evidence of T2 inflammation. These data hint at underlying ontological heterogeneity and suggest that additional pathways may be involved in a subset of patients (2).

Epithelial-derived signals have emerged as critical drivers of asthma severity (5). The interplay between immune and epithelial compartments is now understood to be integral in promoting inflammation and manifesting symptomology associated with severe disease. While prior studies have employed big-data approaches to understand asthma pathogenesis, they have often focused on clinical parameters (6–8) or predefined comparator groups derived from inflammatory phenotype (4, 9–11). Others have utilized asthma-relevant pathway expression from bronchial epithelial cell (BEC) brushings (12) or gene signatures associated with clinically validated biomarkers (13) to cluster patients. Though thought provoking, these analyses often return large lists of genes for further hypothesis testing without prioritization or validation.

Machine learning tools are rapidly evolving and changing the way we approach biological discovery (14). These techniques allow for insights into big data sets that would be difficult or impossible for humans to discern. Machine learning is composed of both unsupervised learning, where algorithms identify inherent structures of unlabeled data, and supervised learning, in which the predictive models may be trained using labeled data (15). To facilitate understanding and improve performance of learning models, feature selection may be utilized to identify elements of a data set most useful for determining an outcome.

In this work, we revisited BEC gene expression data from phases 1 and 2 of the Severe Asthma Research Program (SARP). Prior studies have clustered SARP participants based on BEC expression of genes correlated with fraction exhaled nitric oxide (FeNO) or identified Weighted Gene Coexpression Network Analysis (WGCNA) gene modules associated with clinical characteristics (13, 16). Here, we employed a number of machine learning tools to cluster participants, built a prediction model for external validation, and determined the most useful features (e.g., genes) for classification. Our approach identified heightened IL-18R1 expression in a subset of patients with asthma with impaired lung function and frequent exacerbations. These patients showed transcriptional hallmarks of NF-κB and activator protein 1 (AP-1) activity, despite high levels of corticosteroid (CS) use. We validated IL-18R1 expression and downstream transcription factor (TF) activity at the protein level in the SARP cohort, as well as in patients who underwent video-assisted thoracoscopic surgery (VATS) lung biopsy for severe, treatment refractory asthma.

## Results

### Unsupervised clustering of BEC genes reveals 4 clinically distinct clusters.

A total of 155 participants from the parent SARP 1 and 2 cohort underwent bronchoscopy with bronchial epithelial brushing during phases 1 and 2 of the study. These participants included healthy controls (HCs) and a range of patients with asthma including those with mild to moderate (MMA) and severe asthma (SA) as defined by 2001 ATS guidelines (17). The demographics of this cohort have previously been reported (13).

**Figure 1:**
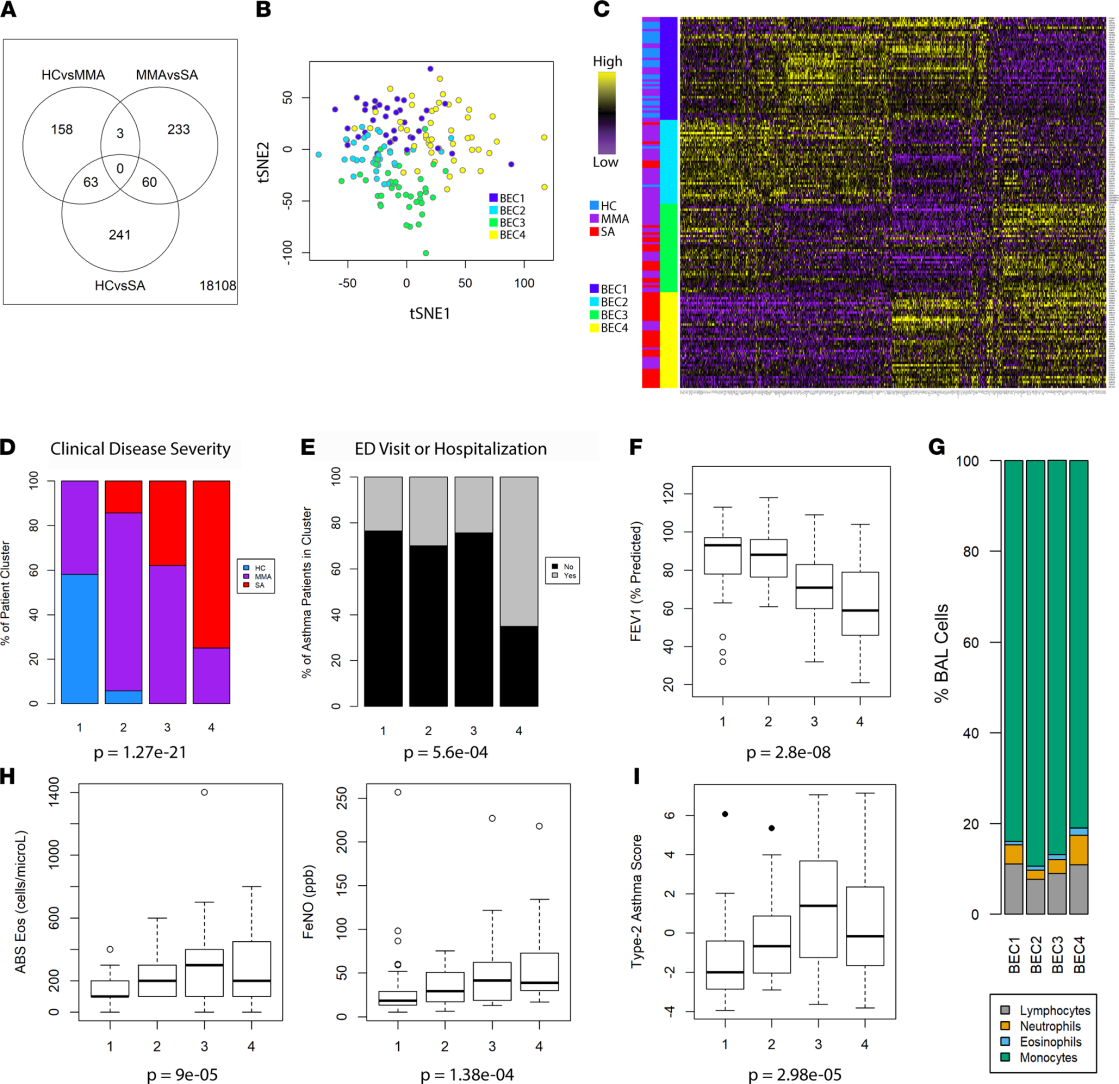
Unsupervised clustering of SARP cohort participants using BEC gene expression. (**A**) Venn diagram of genes differentially expressed between HC, MMA, and SA participants (*n* = 155) after controlling for sex and corticosteroid use. (**B**) Patient clustering results projected on t-stochastic neighbor embedding (tSNE) space. (**C**) Heatmap of expression of the 758 genes included in clustering with patient cluster or clinical disease severity as indicated in row sidebar. (**D**) Clinical disease severity across patient clusters is represented as relative percentage in stacked bar chart with *P* value calculated using Pearson’s χ^2^ testing of raw values. (**E**) Percentage of patients experiencing ED visit or hospitalization for asthma exacerbation in the preceding year is represented as stacked bar chart with *P* value from Pearson’s χ^2^ testing. (**F**) Box plot of FEV_1_ measured by spirometry across patient groups with *P* value calculated using Kruskal-Wallis testing. Error bars represent median values, with bounds of boxes representing IQR and whiskers representing 1.5× the upper or lower IQR. (**G**) Stacked bar plot of BAL cell manual cell count differential across patient clusters. (**H**) T2-biomarkers blood absolute (ABS) eosinophils or fraction exhaled nitric oxide (FeNO) across clusters with *P* value calculated using Kruskal-Wallis testing. (**I**) Geometric mean of Type-2 asthma gene score across patient clusters with *P* value calculated using Kruskal-Wallis testing.

Advances in machine learning offer the opportunity for new insight by returning to richly characterized data sets such as SARP 1 and 2 for reanalysis. As a starting point for our analysis, we identified genes that varied between asthma clinical disease severity classes and HC participants after controlling for biologic sex and CS use. Of 18,108 genes tested, 758 were found to be differential between HC, MMA, and SA participants (Figure 1A). This included transcripts identified as up- and downregulated for each clinical disease severity class, confirming that distinguishing features could be found from a gene expression profile alone. We then used these differentially regulated transcripts for unsupervised participant clustering (Figure 1B). Inspection of gene expression values across patient clusters showed strong intracluster similarity, suggesting favorable performance of our unsupervised learning model (Figure 1C). The 4 patient clusters were approximately similar in size, ranging from 35 to 43 participants (Table 1). We found no difference in race or sex across clusters (Table 1).

Of the patient clusters identified, BEC group 1 (BEC1) housed most of the HCs in the data set, as well as some MMA patients (Figure 1D). The remaining 3 participant clusters were almost exclusively asthma cases, with BEC4 housing ~75% of the patients with SA. Participants in this cluster were slightly older and had higher BMI than in other clusters (Table 1). We found no difference in self-reported allergic symptoms at any time of year, positive skin prick testing, or age of onset across clusters; however, we did find a significantly higher incidence of nasal polyps in BEC4 (Table 1 and Supplemental Figure 1; supplemental material available online with this article; https://doi.org/10.1172/jci.insight.149945DS1). Participants in SA-predominant BEC4 were also more likely to have utilized an acute care setting for asthma care (Figure 1E) and exhibited significantly lower forced expiratory volume in 1 second (FEV_1_), a standard measure of severity in obstructive lung disease (Figure 1F and Supplemental Figure 1).

### Epithelial gene expression better identifies a complex inflammatory phenotype than current T2 biomarkers.

Clinical evaluation of patients with asthma typically involves assessment of inflammatory phenotype, particularly during consideration for molecular targeted therapy in difficult-to-control disease. T2-high (IL-4–, IL-5–, IL-13–driven) asthma is typified by eosinophilic inflammation, while T2-low disease has been linked to neutrophilia and molecular signals such as type 1 IFN and IFN-γ (18). Intriguingly, participants of BEC4 demonstrated a more prominent mixed inflammatory process in their bronchoalveolar lavage (BAL) cells, evidenced by significant enrichment for eosinophils when compared with BEC1 and BEC2 (Dunn’s test adjusted *P* = 0.02 and 0.03) and neutrophils when compared with BEC2 and BEC3 (Dunn’s test adjusted *P* = 0.0006 and 0.007) (Figure 1G and Supplemental Figure 1).

Though the T2 biomarkers were significantly variant across clusters (Figure 1H), BEC3 and BEC4 could not be distinguished by absolute blood eosinophils (post hoc Dunn’s testing *P* = 0.125) or FeNO (post hoc Dunn’s testing *P* = 0.525). These data suggest that, despite differences in transcriptional program, BEC3 and BEC4 would be impossible to differentiate using currently available biomarkers alone. These patient clusters also exhibited similar levels of serum IgE (Supplemental Figure 1). Importantly, BEC3 showed heightened expression of genes associated with epithelial T2 response when compared with others, including BEC4 (post hoc Dunn’s testing *P* = 0.005) (Figure 1I). These data show that participants in BEC3 exhibited a more classical T2 milieu compared with those in BEC4. Taken together, our patient clustering captured a molecular signature associated with very severe, exacerbation prone asthma that was not readily identified by BEC T2 gene expression or standard biomarkers alone.

### OCS dependence is increased in patients with a mixed inflammatory phenotype.

CS are the backbone of asthma treatment and have been shown to have numerous effects on both immune and epithelial cells. We assessed the potential effects of medication compliance across the SARP cohort using BEC expression of the steroid-responsive gene *FKBP5*, observing appropriate increase with escalating doses of inhaled CS (ICS) (Supplemental Figure 2). *FKBP5* expression also increased across patient clusters, consistent with appropriate response to escalating prescription of controller medications. BEC3 and BEC4 showed comparable levels of *FKBP5* expression (post hoc Dunn’s testing *P* = 0.23) and were both significantly increased compared with BEC2 (post hoc Dunn’s testing *P* = 0.03 and 0.015, respectively). These similar levels in BEC3 and BEC4 were observed despite significantly higher levels of oral CS (OCS) use in BEC4 (Supplemental Figure 2). Given the increased OCS use in BEC4, we next investigated whether this may be driving transcriptional changes that identified these subjects. To this end, we performed differential expression analysis between OCS users and nonusers in our data set while controlling for asthma severity and ICS use. We identified 773 significantly variant genes (Supplemental Figure 2). Of these transcripts, only 144 were included in the clustering analysis, suggesting that patient cluster definitions were unlikely to be driven by OCS use alone.

### Pathway analysis identifies patient cluster–specific disease processes.

**Figure 2:**
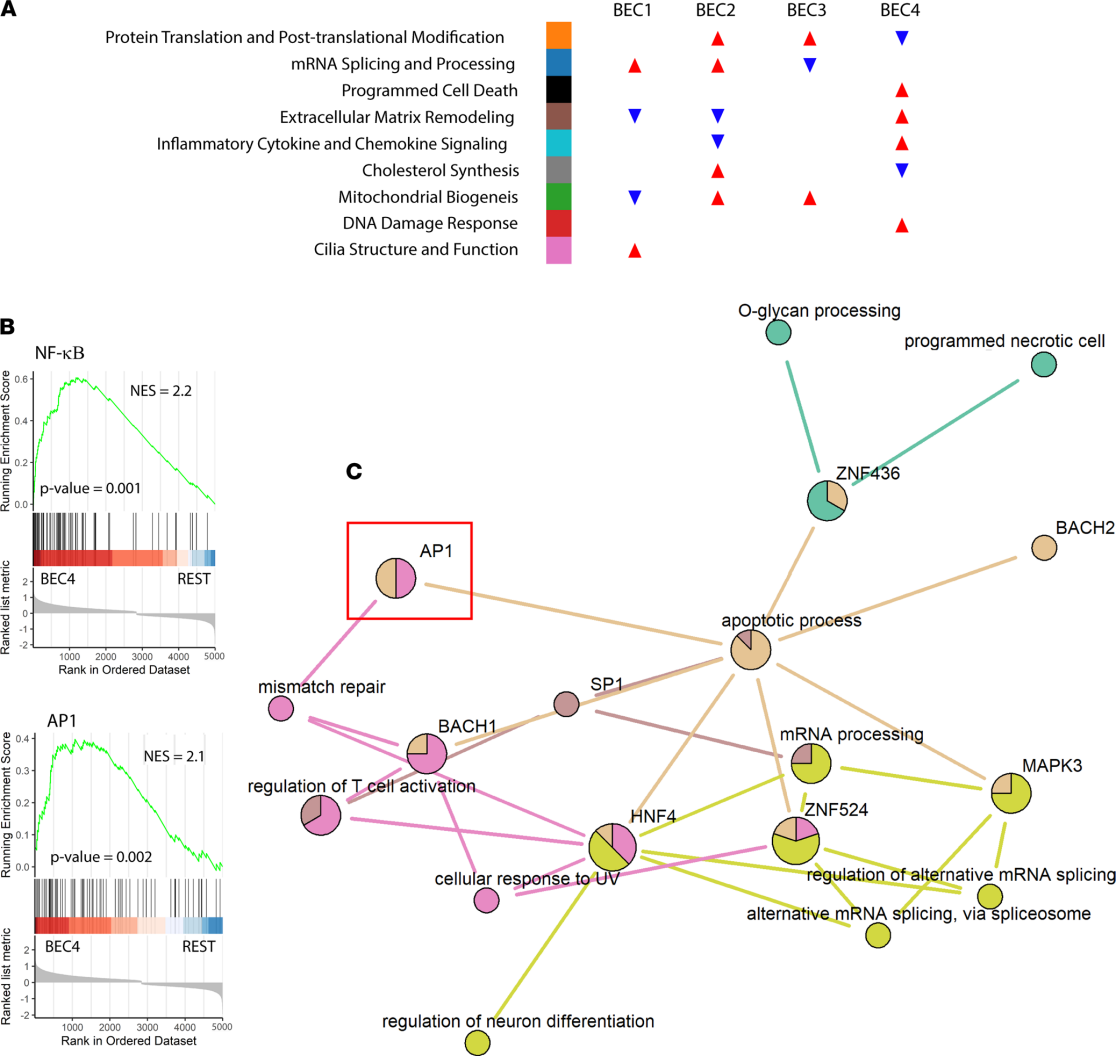
Pathway analysis links cell stress response to AP-1 activity in sick patients with asthma. (**A**) Infographic for summary of Gene Ontology enrichment analysis and Gene Set Enrichment Analysis (GSEA) results as detailed in Supplemental Figures 3 and 4. Color coding next to summary terms is used to indicate relationship to information presented in Supplemental Figures 3 and 4. Arrows indicate relative enrichment (red, upward) or depletion (blue, downward) of genes corresponding to summary terms. (**B**) GSEA results for indicated transcription factor target (TFT) data sets in BEC4 versus remaining SARP participants (REST). *P* values and normalized enrichment scores are shown. (**C**) Network connectivity graph generated by TF2GO illustrates relationships between biological processes (BP) and TFT sets enriched in BEC4 where nodes represent individual BPs or TFTs and edges represent significant overlap in enriched genes as defined by *P* value of hypergeometric overlap < 10^–8^. Edges are colored according to parent BP or TFT, and nodes are colored to demonstrate relative enrichment of connected gene sets.

To better understand the transcriptional programs that defined our patient clusters, we created differential expression models for cluster-specific up- or downregulated genes, accounting for sex and CS use (Supplemental Figure 3). Using these data, we performed gene ontology (GO) enrichment analysis to find biological processes that defined each patient cluster (Supplemental Figure 3). These data were corroborated with Gene Set Enrichment Analysis (GSEA), which relies on different statistical approaches to draw conclusions regarding functionality (Supplemental Figure 4). Patient clusters exhibited distinct biological processes with potential relevance to asthma pathogenesis (Figure 2A). BEC1, which featured most of the HCs in the cohort, showed a relative increase in genes related to cilia structure and function, suggesting deficits in mucociliary clearance as a shared transcriptional feature among asthma-enriched patient clusters. BEC1 also demonstrated relatively lower expression of genes related to extracellular matrix (ECM) remodeling, a feature shared with the well-controlled asthma group BEC2. BEC2 showed lower inflammatory cytokine and chemokine signaling, suggesting appropriate antiinflammatory response to CS therapy in these individuals. Both BEC2 and BEC3 showed similarly higher expression of mitochondrial biogenesis genes compared with other clusters, a process that has been previously implicated in airway disease (19). BEC3 was distinguished by relatively higher ECM remodeling and inflammatory signaling gene expression than BEC2, which may relate to the impairment in lung function, higher CS requirements (Supplemental Figure 2), and elevation of T2 biomarkers seen in BEC3 participants compared with BEC2. Participants in BEC4 exhibited a distinct gene expression profile that included significant enrichment for ECM remodeling and inflammatory signaling when compared with all other clusters. It also featured prominent signals for programmed cell death pathways and the DNA damage response (Figure 2A), supporting a profoundly different transcriptional phenotype in BEC4 compared with all other clusters.

### DNA damage response and programmed cell death are linked to CS refractory AP-1 activation in the most SA patient cluster.

TFs may promote the expression of gene networks responsible for differences observed between our patient clusters. We next used GSEA to identify TFs linked to the gene expression changes (Supplemental Figure 4). Unsupervised ranking for confidence of enrichment identified depletion of NF-κB and AP-1 targets in BEC2 and BEC3 among the most significant TF-related changes between patient clusters. CS have been previously shown to inhibit NF-κB and AP-1 activity, suggesting that participants in these clusters exhibited appropriate response to therapy, as indicated by the decreased expression of target genes. Surprisingly, participants in BEC4 showed markedly heightened expression of NF-κB and AP-1 transcriptional targets (Figure 2B), despite receiving the highest doses of iCS and OCS. These data suggest that subversion of CS response is an intrinsic feature of the most SA patient cluster.

To better understand the relationship between patient cluster–enriched biological processes and TF activation, we developed the R function TF2GO, which constructs networks linking GO terms to TF target sets via commonly shared gene membership (available through Supplemental Data Set 1 and online at https://github.com/camiolomj/TF2GO/). Using TF2GO, we constructed network maps for each of the patient clusters based on upregulated genes from differential expression analysis (Figure 2C and Supplemental Figure 5). Retinoic acid–related orphan receptor-α (RORα) was linked to mitochondrial biogenesis in BEC2 and BEC3 (Supplemental Figure 5), consistent with previous reports supporting its role in mitochondrial quality (20). Interestingly, RORα was also shown to promote epithelial integrity via attenuation of NF-κB transcriptional activity, suggesting that it may play multiple roles in asthma severity (21). Programmed cell death and DNA damage response were specifically enriched in BEC4 (Figure 2A). We found linkage between the AP-1 regulatory network and these processes, specifically in BEC4 participants (Figure 2C). These data suggest that paradoxical activation of AP-1 target genes, despite high doses of ICS or even chronic OCS use, may promote the distinguishing biological features of BECs from the most severe patient cluster.

### Machine learning identifies a 31-gene signature that recapitulates and validates patient clustering.

**Figure 3:**
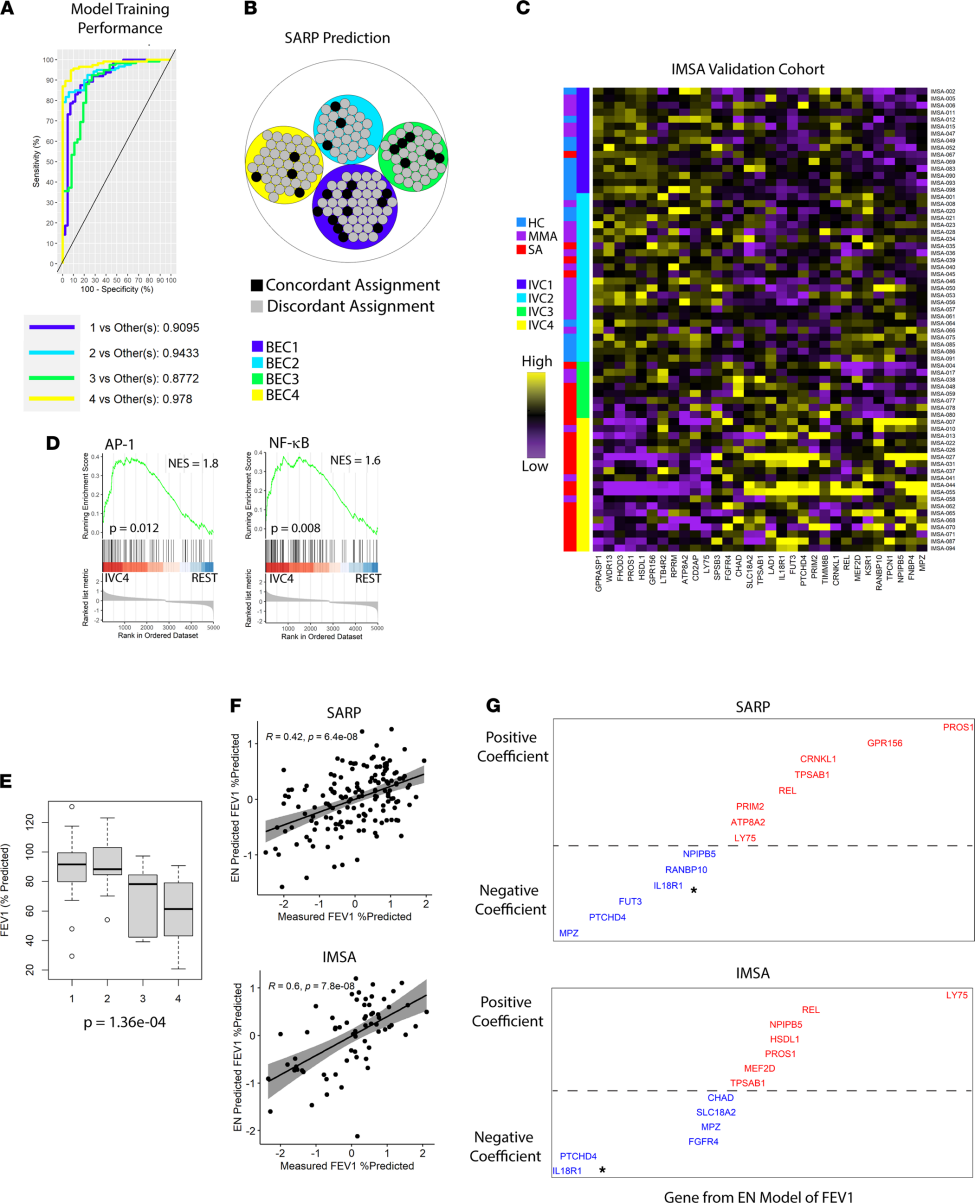
Machine learning validation of a 31-gene signature for patient clustering. (**A**) Receiver operating characteristics (ROC) curve of a sparse-partial least squares discriminant analysis (sPLS-DA) model training for k-means cluster prediction on the SARP cohort. ROC curves were calculated as one class versus the others using 5 fold-validation on the original training set. Reported AUC are based on comparison of predicted scores of one class versus the others. (**B**) Circle plot demonstrating concordance of assignment between original SARP clustering and 31-gene solution. (**C**) Heatmap of expression of the genes included in clustering of the IMSA validation cohort with patient cluster or clinical disease severity as indicated in row sidebar. (**D**) GSEA results for indicated transcription factor target datasets in IVC4 vs remaining IMSA participants (REST). (**E**) Box plot of FEV_1_ across IMSA clusters with *P* value from Kruskal-Wallis testing. Error bars represent medians, with bounds of boxes representing IQR and whiskers representing 1.5× the upper or lower IQR. (**F**) Elastic net–predicted (EN-predicted) FEV_1_ based on gene expression versus measured FEV_1_% predicted in SARP or IMSA. Grayed area indicates the 95% confidence bounds around a linear regression model comparing the 2. Spearman’s ρ and *P* value are shown. (**G**) Graphical representation of EN modeling determinants of lung function (FEV_1_). Coefficients from SARP or IMSA are plotted in order of ascending value from left to right, with distance from the hashed line indicating magnitude of contribution to the model. Blue coloration of transcript ID denotes a negative coefficient, and red indicates positive. Asterisk in plot areas denote IL-18R1 in either data set.

Candidate selection from large patient sets is often constrained by the complex nature of the data produced from the analysis. Satisfied that our clustering had identified clinically and molecularly interesting phenotypes, we next utilized supervised learning and feature selection to identify a paired-down list of candidate genes of interest (Supplemental Figure 6). To do so, we first selected genes with greatest weighting from principal component analysis (PCA), as prior studies have demonstrated similarity between PCA and k-means clustering (22). For details, please see the R script “Supervised Patient Classification” in Supplemental Data Set 1. Using these varying gene lists derived from eigenvectors identified by PCA, we trained a supervised learning model for classification on the original SARP data set. Plotting out model error rate, we identified a solution that provided the most effective patient clustering while utilizing the least number of features (Supplemental Figure 6). This model showed strong performance on the original SARP data set (Figure 3A), achieving a concordance of assignment > 85% (Figure 3B). In all, 31 genes were required for our supervised prediction model.

To test the external validity of our gene signature, we turned to a separate cohort of HCs, MMAs, and patients with SA with bronchoscopically obtained airway brushings. The Immune Mechanisms of Severe Asthma (IMSA) cohort was enrolled nearly a decade after SARP 1 and 2, shared no participant with the earlier study, and utilized RNA sequencing as opposed to microarray technology (23). We used our supervised learning model to predict patient cluster in the IMSA cohort, identifying 4 clusters with similar characteristics to those identified in SARP. IMSA validation cluster-1 (IVC1) was again composed of mostly HCs and MMA patients (Figure 3C). The other 3 clusters were enriched for asthma cases, with IVC4 being almost exclusively SA. IMSA patient clusters demonstrated significant difference in OCS use (*P* = 0.009) and acute care utilization for asthma (*P* = 0.002), mirroring SARP data. GSEA again showed enrichment for NF-κB and AP-1 transcriptional targets in IVC4, despite high levels of CS exposure, recapitulating this crucial finding from SARP (Figure 3D). 

Our validation patient clusters displayed significant decrement to lung function (Figure 3E), with IVC4 showing the greatest impairment FEV_1_. Since our pathway analysis data demonstrate increased extracellular remodeling, inflammatory signaling, and cell death in this patient cluster, we reasoned that loss in FEV_1_ may linked to these processes. We next employed elastic net (EN) modeling of FEV_1_ on both SARP and IMSA cohorts using the gene signature identified by our supervised learning model. Our EN models of FEV_1_ prediction proved highly effective in both cohorts (Figure 3F), confirming an intrinsic link between lung function and transcripts critical to our patient clustering. Multiple coefficients of determination were shared between cohorts and were able to be validated using Spearman’s rank correlation of FEV_1_ versus gene expression value (Figure 3G and Supplemental Figure 7). Among these, IL-18 receptor 1 (IL-18R1) stood out as a strong negative predictor of FEV_1_ in both cohorts (Figure 3G). Critically, IL-18 has been described as an upstream activator of both NF-κB and AP-1 pathways, suggesting a possible link between transcriptional hallmarks of BEC4/IVC4 and this signaling axis (24, 25).

### IL-18R1 expression is increased in the most SA patient clusters and tied to both T1 and T2 immune pathways.

**Figure 4:**
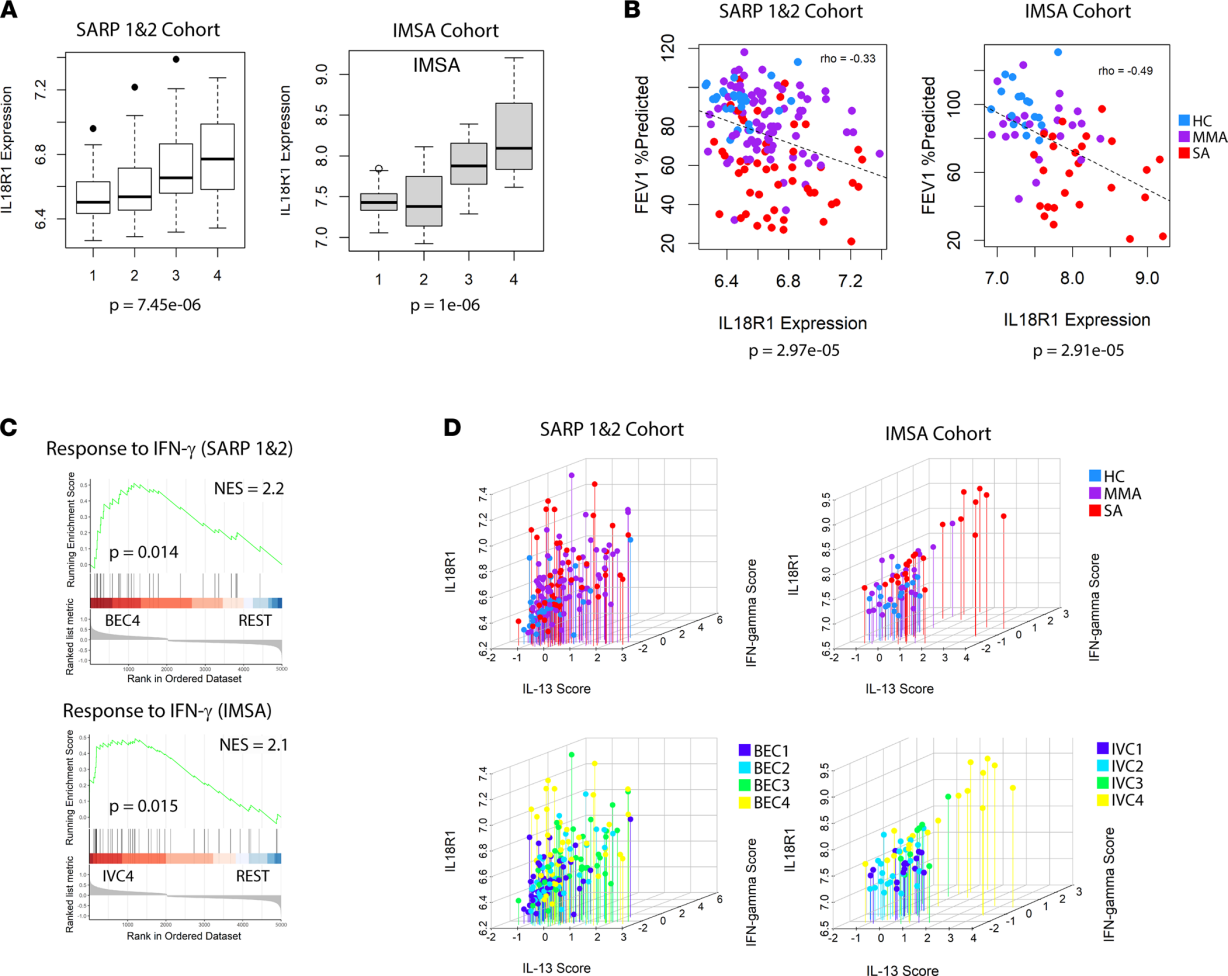
IL-18R1 expression is negatively associated with lung function and linked to mixed inflammation. (**A**) IL-18R1 expression across patient clusters in the SARP or IMSA cohorts. Error bars represent median values, with bounds of boxes representing IQR and whiskers representing 1.5× the upper or lower IQR. (**B**) Plot of FEV_1_% predicted versus IL-18R1 expression in the SARP or IMSA cohorts. Hashed line represents a linear regression model comparing them. Spearman’s ρ and *P* value are indicated. Data points are colored according to clinical disease severity. (**C**) GSEA results for IFN-γ response in BEC4 versus remaining SARP participants (REST) or IVC4 versus remaining IMSA participants (REST). (**D**) Geometric mean IL-13 signature expression score, geometric mean IFN-γ signature expression score and IL-18R1 expression plotted for participants of the SARP or IMSA cohorts. Individuals are colored by either clinical disease severity (top) or patient clustering result (bottom).

IL-18R1 showed strong negative association with FEV_1_ in our EN model of lung function and was a component of our 31-gene signature for patient classification, suggesting its expression may have critical function in promoting a SA phenotype. It is also a potentially targetable protein via neutralizing antibodies to cytokine or receptor moieties. IL-18R1 is found in the asthma susceptibility locus on chromosome 2q12 and has been linked to asthma risk in genome-wide association studies (26, 27). Prior evidence suggests that expression of IL-18 pathway components, specifically IL-18R1, differs between those with asthma and those without (28–30). We found that IL-18R1 transcript levels were highest in SARP BEC4, as well as IMSA IVC4 (Figure 4A). IL-18R1 was also negatively correlated with FEV_1_ in both SARP and IMSA cohorts (Figure 4B).

IL-18 was initially described as an IFN-γ–inducing factor that was later found to also promote T2 inflammation in the absence of IL-12 (31, 32). Using GSEA, we found enrichment of IFN-γ response genes in BEC4 and IVC4, suggesting that IL-18 signaling may indeed be active in these individuals (Figure 4C). BAL cells from IL-18R1^hi^ patient clusters demonstrated mixed eosinophilia and neutrophilia, suggestive of a mixed inflammatory response. Using in vitro cytokine stimulation data mined from the Gene Expression Omnibus (GEO; http://www.ncbi.nlm.nih.gov/geo/), we compared IL-18R1 expression level with scoring of gene expression associated with for IL-13 and IFN-γ treatment conditions (Figure 4D). We observed correspondence between IL-18R1 expression, mixed Type-1 (T1) and T2 inflammatory state, and clinical disease severity in both asthma cohorts.

### IL-18 pathway components are increased in the most severe asthma patients.

**Figure 5:**
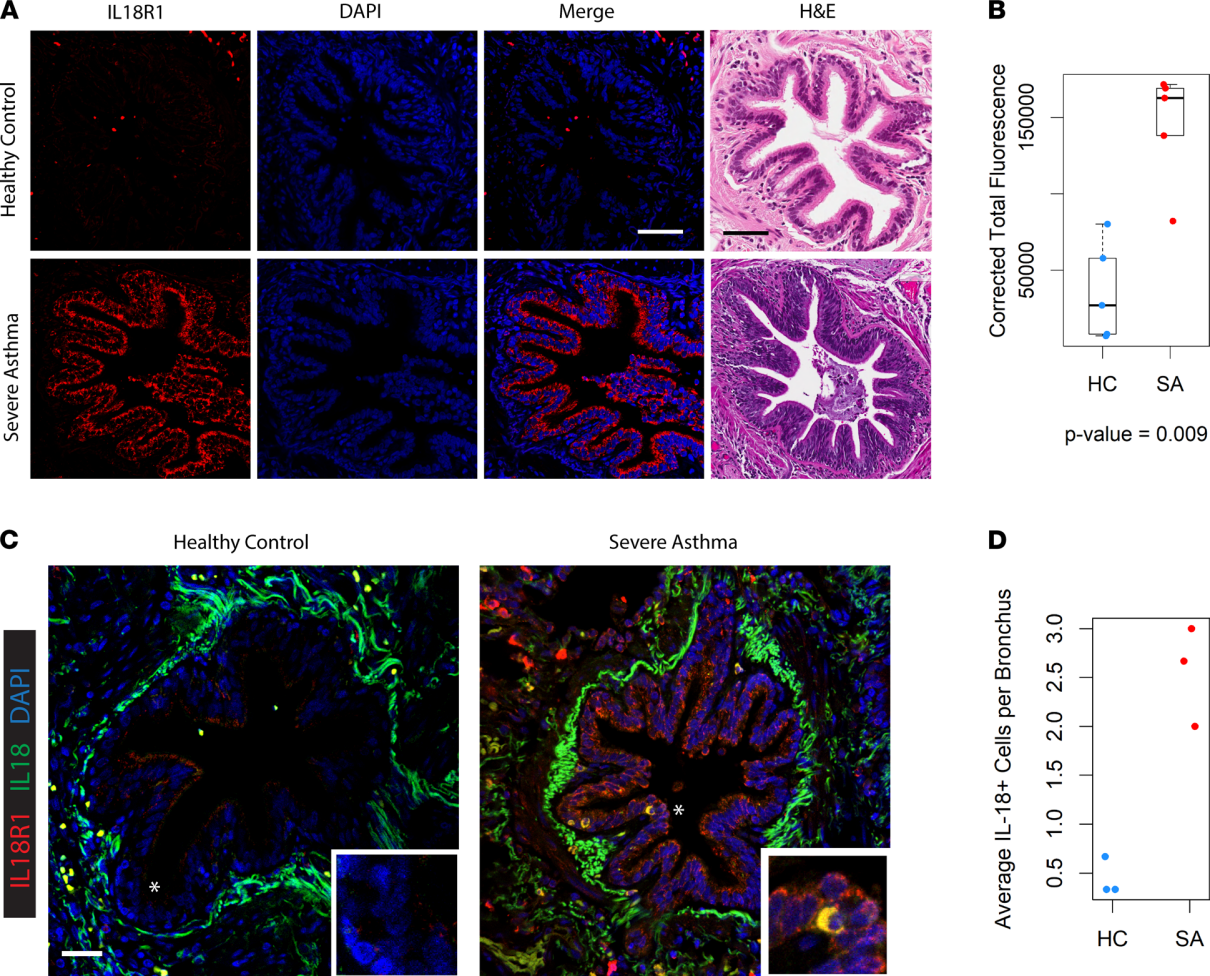
IL-18R1 expression and IL-18^+^ cells are increased in the distal bronchi of patients with SA. (**A**) Immunofluorescence (IF) microscopy of VATS biopsies from healthy controls and patients with SA. Representative fields shown from an assessment of *n* = 5 subjects from each group. Scale bar: 300 μm. H&E staining of serial sections of respective airways are illustrated next to IF images. (**B**) Quantification of immunofluorescence labeling of IL-18R1 corrected for background signal. Individual points represent mean value from (*n* = 3) technical replicates of *n* = 5 samples for HC and SA comparison groups. *P* value calculated from Wilcoxan signed-rank test. Error bars represent median values, with bounds of boxes representing IQR and whiskers representing 1.5× the upper and lower IQR. (**C**) Staining for IL-18R1 and IL-18 in the airways of HCs and patients with SA demonstrates increased presence of double-positive cells within SAs compared with HCs. Asterisk denotes region of inset. Scale bar: 300 μm. Representative field shown for *n* = 3 subjects for each group. (**D**) Quantification of IL-18^+^ cells per distal bronchus in HCs and patients with SA (*n* = 3 for each group). Individual points represent mean value from (*n* = 3) technical replicates.

Given these suggestive RNA data, we validated the protein expression of IL-18R1 using a separate cohort of VATS lung biopsies in patients with extremely severe asthma who underwent the procedure to rule out confounding diagnoses such as eosinophilic granulomatosis with polyangiitis. IF microscopy of their distal airway tissue demonstrated greater staining for IL-18R1 as compared with HC tissue obtained from spontaneous pneumothorax patients (Figure 5A), supporting an increase in IL-18R1 at both the transcript and protein level in both distal and proximal bronchial epithelium of patients with SA. Quantification of staining (*n* = 5 samples for each) showed greater than a 3-fold median increase of IL-18R1 expression in the airways of the most SA patients (Figure 5B). Having found increased IL-18R1 expression in SA, we verified the presence of cellular sources of IL-18 in the airway (Figure 5C). IF microscopy verified increased number of IL-18^+^ cells in patients with SA compared with HC (Figure 5D). Of note, IL-18R1^+^ and IL-18^+^ double-positive cells could be identified in the epithelial and lamina propria layers of both patients with SA and HCs, though the identity of these dual-expressing cells has yet to be determined. Our tissue validation analysis confirmed protein-level changes in IL-18R1 expression among the most SA patients and identified cellular sources of IL-18 as increased in their airways.

### Activation of downstream NF-κB and AP-1 is present in IL-18R1^hi^ patients with SA.

Previous study of IL-18 has implicated both NF-κB and AP-1 in downstream transduction following receptor engagement (24, 25). Participant clustering from both the SARP and IMSA cohorts demonstrated that IL-18R1^hi^ individuals exhibited enrichment of transcriptional targets of NF-κB and AP-1. We next sought to verify pathway activation using staining of samples from the original cohort.

**Figure 6:**
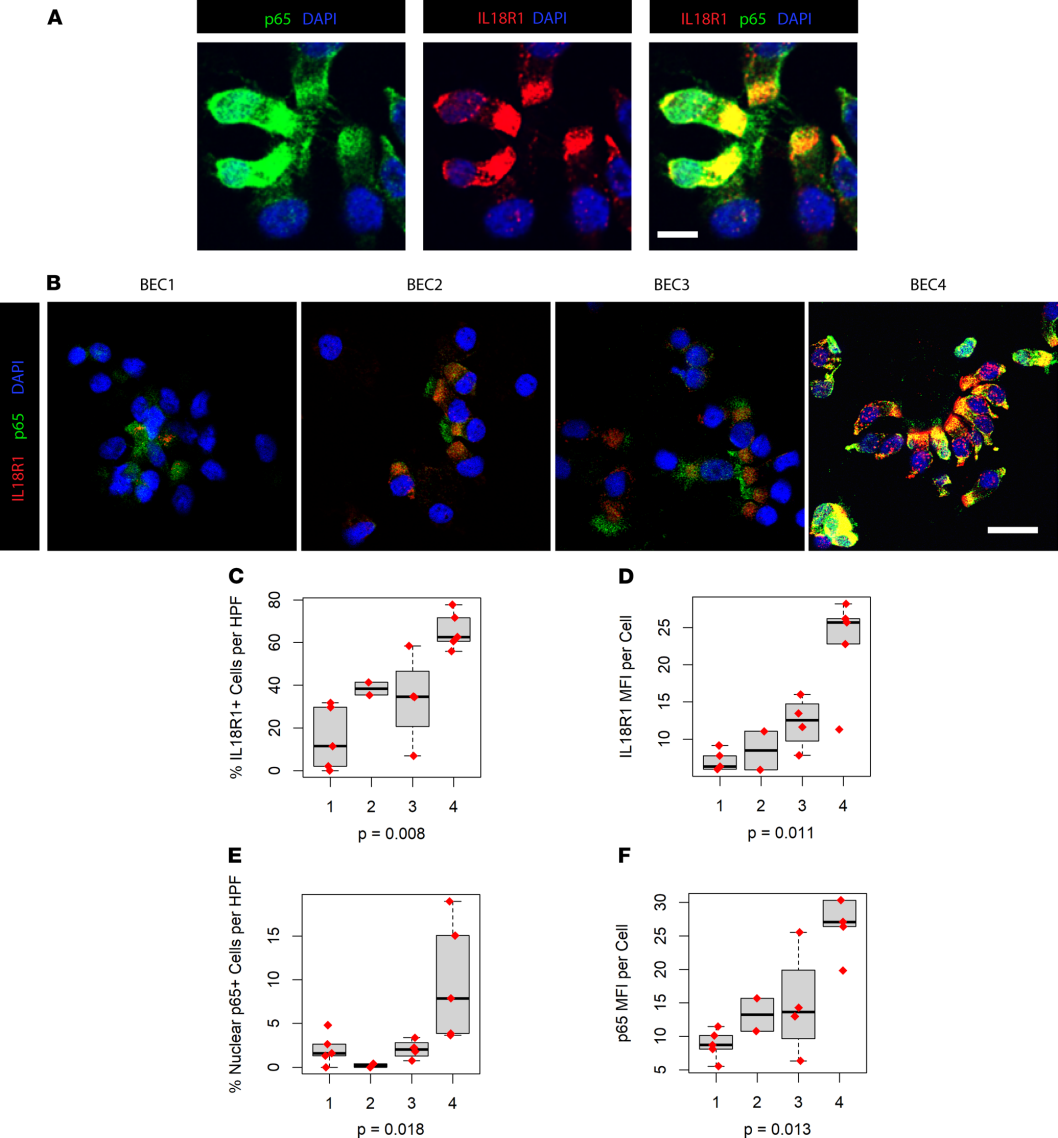
IL-18R1^hi^ patients exhibit Iincreased nuclear translocation of NF-κB family member p65. (**A**) Immunofluorescence (IF) microscopy of cytospin preparations from endobronchial brushings of the SARP cohort demonstrating nuclear translocation of p65 in IL-18R1^+^ epithelium. (**B**) Representative fields from IF staining of cytospins in the indicated participant clusters from SARP. (**C**) Quantification of percent IL-18R1^+^ cells per HPF, expressed as percentage of DAPI^+^ nuclei. Scale bar: 25 μm. Total *n* = 15. *P* value calculated from Kruskal-Wallis test. Error bars represent median values, with bounds of boxes representing IQR and whiskers representing 1.5× the upper and lower IQR. (**D**) Mean fluorescence intensity (MFI) of IL-18R1^+^ cells from each patient cluster, with significance calculated by Kruskal-Wallis. (**E**) Quantification of percent cells with nuclear translocation of p65 per HPF, expressed as percentage of DAP^+^ nuclei, with significance calculated by Kruskal-Wallis. (**F**) MFI of p65^+^ cells from each patient cluster, with significance calculated by Kruskal-Wallis.

NF-κB TFs are master regulators of immune and inflammatory response. In the latent state, they are sequestered in the cytosol by their inhibitor IκB (inhibitor of NF-κB). Upon stimulation of upstream receptors, a series of events leads to proteasomal degradation of IκBs and release of NF-κB for nuclear translocation and activation of gene transcription (33). The NF-κB family of TFs consists of 5 members, p50, p52, p65 (RelA), c-Rel, and RelB, though the transcription activation domain necessary for positive regulation of gene expression is present only in p65, c-Rel, and RelB. We stained cytospin preparations from epithelial brushings of participants from the SARP cohort for p65 and were able to identify cells with evidence of nuclear translocation (Figure 6A). Assaying participants from our unsupervised transcriptional clusters (Figure 6B), we saw increased IL-18R1 staining and nuclear translocation of p65 as predicted by pathway analysis. Quantification of IL-18R1^+^ cell per high-powered field (HPF) (Figure 6C), as well as mean fluorescence intensity (MFI) of IL-18R1 staining (Figure 6D), showed that BEC4 participants had the greatest number of IL-18R1^+^ cells, as well as the greatest magnitude of IL-18R1 production per cell. BEC4 participants also showed the greatest percentage of cells with nuclear translocation of p65 (Figure 6E) and greatest MFI of p65 (Figure 6F).

**Figure 7:**
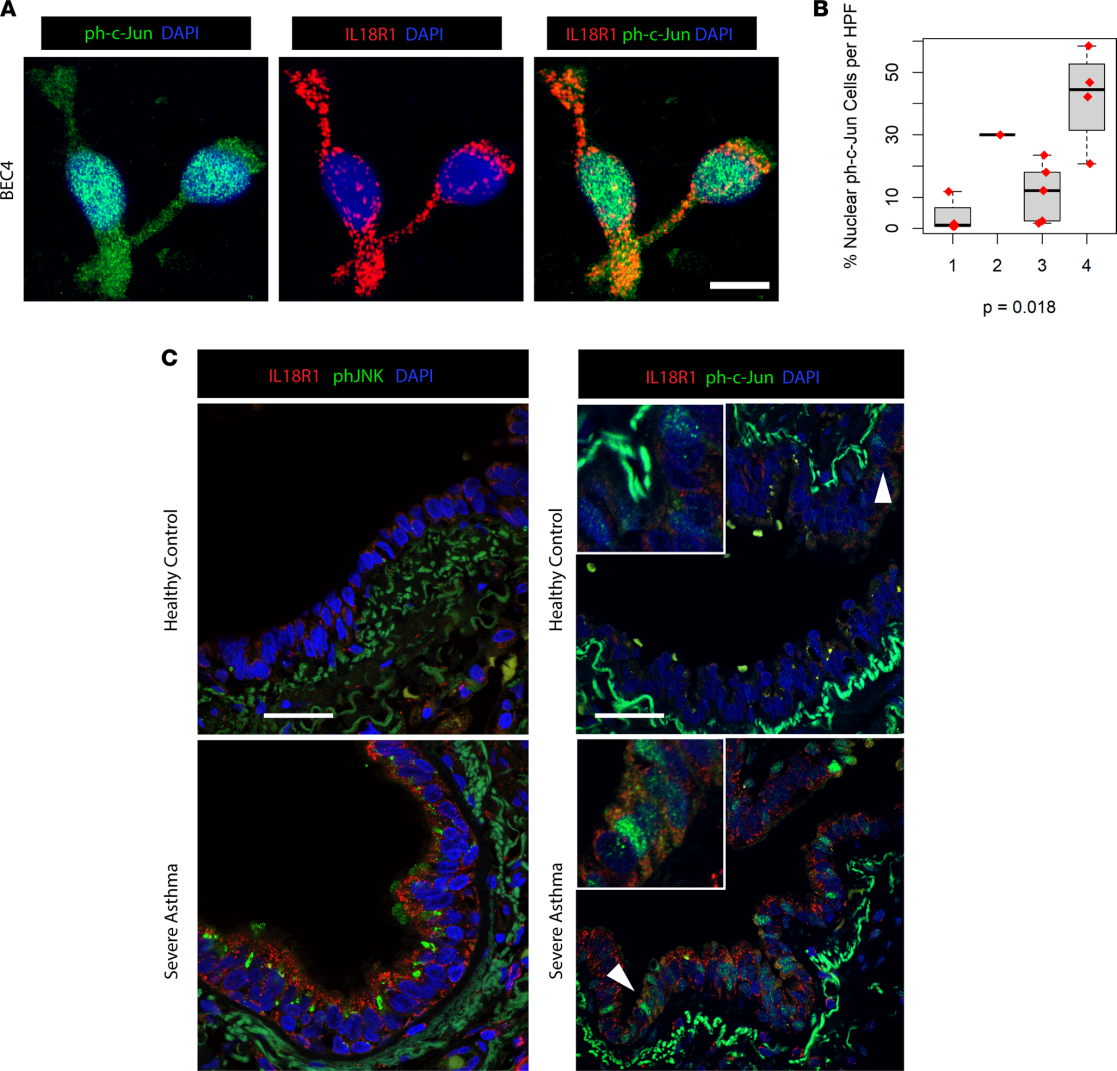
AP-1 pathway activation is present in IL-18R1^hi^ SA airways. (**A**) Immunofluorescence (IF) microscopy of cytospin preparations from endobronchial brushings of the SARP cohort demonstrating nuclear translocation of phosphorylated c-Jun (ph–c-Jun) in IL-18R1^+^ epithelium. (**B**) Quantification of percent cells with nuclear translocation of ph–c-Jun per HPF, expressed as percentage of DAPI^+^ nuclei. Scale bar: 25 μm. Total *n* = 14. *P* value calculated from Kruskal-Wallis test. Error bars represent median values, with bounds of boxes representing IQR and whiskers representing 1.5× the upper and lower IQR. (**C**) Staining for phosphorylated JNK (phJNK) or phosphorylated c-Jun (ph–c-Jun) in the airways of HCs and SAs. Scale bar: 150 μm. Representative field shown for *n* = 5 subjects for each group.

Previous work on IL-18 signaling in lung epithelial cells identified AP-1 transactivation as a downstream effector mechanism (34). AP-1 is a heterodimer composed of proteins belonging to the c-Fos, c-Jun, JDP, and ATF families (35). Notably, our IL-18R1^hi^ patient clusters demonstrated activation of the AP-1 TF network despite receiving the highest levels of CS exposure in the cohort. We stained additional cytospins from epithelial brushings of participants from the SARP cohort for the phosphorylated, active form of c-Jun (ph–c-Jun) and identified IL-18R1^+^ cells with evidence of nuclear translocation (Figure 7A). Quantification showed significantly greater ph–c-Jun^+^ cells per HPF in BEC4 participants, corroborating TF target analysis (Figure 7B). Supporting this finding, high levels of the active, ph–c-Jun N-terminal Kinase (phJNK), a known downstream target of IL-18R1, were detected in the cytosol of SA but not HC BECs from our VATS biopsy cohort (Figure 7C). We also observed ph–c-Jun within the cytosol and nucleus of distal lung BECs from SA but not HC tissues (Figure 7C). These findings confirm transcriptional analysis showing enrichment of NF-κB and AP-1 targets in IL-18R1^hi^ patients. As both pathways are known to be negatively regulated via glucocorticoid signaling (36, 37), these data again suggest subversion of CS responses in IL-18R1^hi^ patients with asthma, despite treatment with high-dose CS.

## Discussion

Using a combination of machine learning tools for reanalysis of the SARP 1 and 2 cohort, we identified the targetable IL-18 pathway from over 18,000 genes expressed by airway epithelial cells. We began by identifying differentially expressed transcripts across asthma severity and then utilized a combination of unsupervised clustering, feature selection, and supervised learning to identify a 31-gene signature able to capture important clinical and molecular phenotypes. This signature was then validated on the external IMSA cohort, reconstituting patient clusters with remarkable similarity to those in SARP. Our approach to identifying candidate genes of interest combined this signature with lung-function modeling, a critical clinical characteristic that varied between clusters. Importantly, though lung function may play a significant role in how a patient experiences morbidity from asthma, low lung function is not essential for the diagnosis of severe disease. Therefore, capturing this phenotype was less likely a consequence of our initial approach to gene filtering.

Using both biased and unbiased approaches, multiple asthma phenotypes have been previously described. Analysis of the Unbiased Biomarkers in Prediction of Respiratory Disease Outcomes (U-BIOPRED) cohort utilized patient clustering based on gene set variation analysis (GSVA) signatures from bronchial biopsies and brushings to identify T2^hi^ and T2^lo^ patients (12). Unlike our study, this work utilized preselected gene sets derived from literature on asthma pathobiology, potentially limiting discovery. Previous clustering of the SARP cohort used genes correlated with FeNO to find expression patterns associated with varying clinical features, including lower lung function, atopy, eosinophilic inflammation, and age of onset (13). Our study employs different methodology to refine this analysis, including a variety of systems-level techniques to infer NF-κB and AP-1 activity in IL-18R1^hi^ patients, which is then validated in multiple external cohorts.

The patient clusters identified in our study varied across a number of parameters, including BMI, age, and lung function (Table 1). BEC1, which housed the vast majority of HCs, had lower levels of the T2 biomarkers FeNO and absolute blood eosinophils than the 3 asthma-predominant clusters. Of these clusters, BEC2 had relatively intact lung function and could be distinguished at the molecular level by relative downregulation of genes associated with inflammatory cytokine signaling and ECM remodeling. Of the remaining 2 asthma clusters, BEC3 showed very high T2 gene expression and relatively worse lung function when compared with BEC1 and BEC2. BEC4 exhibited mixed neutrophilic and eosinophilic inflammation in BAL, had the worst lung function across cohort participants, and had the highest burden of chronic OCS use.

BEC4, identified as the most severe cluster, was highly distinguishable from other asthma clusters by its unique transcriptional profile. There was evidence for activation of cell death pathways, despite study participants being free of exacerbation or infection at the time of bronchoscopy. Thus, BECs of these individuals may be primed for overexuberant stress responses or experience chronic cell death even in steady state conditions. Recent work from our laboratory and others has demonstrated a role for nonapoptotic programmed cell death in asthma bronchial epithelium (38, 39). Chronic activation of these pathways could be the cause or consequence of chronic inflammation — and even asthma exacerbations themselves.

Earlier gene expression studies have identified T2 immune, epithelial growth and repair, inflammasome activity, and CS-related genes in relation to more SA (12, 13, 16) but with little ability to distinguish the most relevant genes or pathways. Here, we have employed feature selection from PCA analysis to generate candidate gene lists for supervised model training. This process allowed us to focus on principal sources of variance in the data set based on the assumption that these genes may be of biologic significance. The performance of our supervised model system was objectively measured in relation to the number of genes required, with the goal of selecting parameters that optimized classification accuracy while still allowing us to focus on a small, manageable subset of the original gene pool. We, thus, identified 31 transcripts able to cluster the SARP cohort, with 85% concordance to the assignment initially achieved with over 700 genes. Subsequent EN modeling of FEV_1_ narrowed that list to a handful of candidates and highlighted the potential importance of IL-18R1. The coexpression of TF networks downstream of IL-18R1 during pathway analysis further guided our study.

IL-18R1^hi^ patient clusters in both the SARP and IMSA cohorts showed enrichment for AP-1 transcriptional targets; AP-1 is a known downstream target of IL-18 signaling in human bronchial epithelium (34). Our study, therefore, identifies the IL-18RI/JNK/AP-1 axis as a potentially critical inflammatory pathway linked to the most SA. Our computational identification is supported by numerous previous studies linking epithelial IL-18R1 expression to more SA (13, 16, 40) and the consistent linkage of single nucleotide polymorphisms in the IL-18R1 genetic locus to asthma (41). To further investigate this pathway, we confirmed expression of IL-18R1 on the bronchial epithelium of the most SA patients, as well as the presence of IL-18–producing cells within and adjacent to their airways. In fact, the cytokine, its receptor, and evidence of downstream JNK/AP-1 pathway activation were all concomitantly observed. Importantly, we also identified IL-18^+^ and IL-18R1^+^ double-positive cells in the airways of patients with SA, suggesting that signaling may occur in an autocrine manner consistent with evidence suggesting IL-18 promotes expression of its own receptor via a positive feedback loop (42).

IL-18’s role in asthma or its phenotypes remains speculative, partly because it is a complex cytokine that may promote either T1 or T2 inflammation depending on the presence or absence of IL-12 (43). BEC4 showed evidence of both T1 and T2 immunity, with mixed eosinophilic and neutrophilic infiltrates in BAL, elevation of T2 biomarkers, and activation of IFN response pathways at the transcriptional level. IL-18 may promote type I inflammation via immune cell production of IFN-γ (44, 45), which has been previously implicated in CS-resistant asthma (2, 46–50). Additionally, IFN signaling, a hallmark of viral infection, is heightened in subsets of patients with SA (23). In humans, IFN-γ has been linked to neutrophil chemotaxis via the chemokine receptors CCR1 and CCR3 (51).

In our recent work with the IMSA cohort, we identified a subset of patients with SA characterized by enrichment of IFN-γ^+^ T cells in their BAL fluid in whom IL-18 may be an upstream regulator (52). It is important to note that these patients also displayed concomitant T2 inflammation, which was indistinguishable from those without the high T1 phenotype. In the present study, patients with hallmarks of IL-18/AP-1 activation were also poorly distinguished by peripheral markers of T2 inflammation, suggesting that T2 inflammation runs in parallel or is differently regulated across BEC3 and BEC4. It is notable that, in the absence of IL-12, IL-18 has been reported to drive IL-4 production from basophils or NKT cells, consistent with differing sources of T2 cytokines across clusters (53–55). Thus, IL-18 could promote T2 cytokine production from innate cells or lymphocytes in patients with SA who do not harbor a distinctly T1^hi^ phenotype.

IL-18 signaling is influenced by multiple factors, including the level of free protein unbound by its endogenous inhibitor, IL-18 binding protein (IL-18BP), known to be in BAL fluid (56, 57). Once the receptor is activated, signal transduction is accomplished via molecules including MyD88, IRAKs, and TRAF6 leading to activation of the NF-κB or AP-1 TFs (58). Costimulation with IL-12, which leads to STAT4 activation, may further promote expression of specific genes in T cells such, as *Ifng*, through combinatorial effects on its promoter region (43). Cell lineage may also determine the final impact of IL-18. While IL-1β induced the expression of the NF-κB reporter genes, in human lung epithelial cells, IL-18 responses were weak or absent (34). IL-18 treatment did, however, cause significant transactivation of AP-1 reporter constructs (34). Given the persistent activation of NF- κB targets in IL-18R1^hi^ patient clusters, however, the influence of concomitant signaling on downstream IL-18 transduction must be explored in future studies.

AP-1 pathway activation has a well-established role in cellular stress response (35). As IL-18 is an inflammasome-activated protein, paracrine signaling via this axis may play an important role in mediating epithelial stress in SA. Furthermore, NF-κB activation may reciprocally promote inflammasome-mediated signaling via transcription of key constituents required for cleavage and activation of IL-18 (59). IL-18R1 showed positive correlation to gasdermin-B (GSDMB, *P* = 2.4 × 10^–6^, ρ = 0.55) and GSDMC (*P* < 2.2 × 10^–16^, ρ = 0.67) in our study. GSDMB was recently implicated in pyroptosis of human embryonic kidney epithelial cells (60). Thus, IL-18 signaling evident in BEC4 may lead to pyroptosis of airway epithelial cells via noncanonical mechanisms involving other gasdermin family members such as GSDMB and GSDMC.

Despite the inflammatory and death signals found in BEC4, patients in this cluster are on the highest doses of CS. CS regulate gene expression by binding to the glucocorticoid receptor (GR) causing the ligand-bound receptor to translocate to the nucleus (61). A central mechanism by which CS exert antiinflammatory effects is via transrepression of AP-1 and NF-κB by ligand-bound GR (37, 62, 63) resulting in downregulation of their targets (64–66). Previous work has demonstrated increased levels of active, phosphorylated JNK, and downstream AP-1 in the peripheral blood cells and bronchial epithelium of patients with CS-resistant asthma (67, 68). Direct phosphorylation of GR at S226 by active JNK was linked to nuclear export of GR, thus abrogating its impact on gene expression (69). IL-18–induced JNK activation could, therefor, interfere with CS response via GR S226 phosphorylation, leading to unimpeded transactivation of target genes AP-1 and NF-κB as observed in BEC4.

In summary, using machine learning, we identified and then validated a transcriptional signature that discerned a cluster of patients with high CS dosing, frequent exacerbations, and impaired lung function. Using additional downstream analysis, we associated the most severe and physiologically impaired patients with high levels of IL-18R1 expression. This expression was associated with persistent NF-κB, AP-1, ECM remodeling, and cell death pathway activation, as well as poor response to high-dose CS. These findings strongly support investigation of IL-18 as a therapeutic target in CS-refractory disease.

## Methods

### Materials availability.

Software and custom code can be found in Supplemental Methods, as well as online at: https://github.com/camiolomj/TF2GO

### Human cohort gene expression data availability.

The SARP microarray data set is available online in the National Center for Biotechnology Information’s GEO database (accession GSE63142 and GSE43696). The IMSA RNA sequencing data set is available through GEO accession GSE158752.

### SARP patient clustering.

Microarray gene expression data was prepared as previously described (13). Briefly, microarray images were processed according to Agilent Feature Extraction protocol in 3 batches. Normalization was done using cyclic-LOESS, and floor value was set as the mean signal across all negative control probes, across all samples. Differential expression between HC, MMA, and patients with SA was performed using the limma package for R (70) (version 3.46.0) accounting for CS use and sex. Genes were filtered for significance using a threshold *P* value of less than 0.05 after correction for a FDR less than 5%. For details, please see Disease Severity DEG in Supplemental Methods. The determination of 4 patient groups was arrived at by gap statistic calculation using the R package “NbClust” (71) (version 3.0). Clustering was performed using the R package “multiClust” implementation of k-means (72) (version 1.16.0). For details, please see Participant Clustering and DEG in Supplemental Methods.

### Epithelial phenotype scoring.

Patients in SARP were scored for strength of expression for a curated list of genes related to T2 asthma phenotype that includes CLCA1, POSTN, and SERPINB2 (4). Mean value of these 3 genes were calculated for each cohort participant.

### Differential expression analysis.

For OCS user versus nonuser analysis, differential expression was performed using the limma package for R accounting for ICS use and sex (70). To identify patient cluster–specific transcript, the limma package for R was used accounting for CS use and sex. Patient cluster–specific up- or downregulated transcripts were determined based on significant difference in expression to all 3 other groups as defined by a *P* value of less than 0.05 after correction for FDR < 5%. For details, please see Participant Clustering and DEG in Supplemental Methods.

### GO analysis.

GO enrichment analysis was performed using the TopGO package for R (73) (version 2.42.0). Patient cluster–specific downregulated processes were determined using term enrichment from patient cluster–specific downregulated genes, as identified above. Patient cluster–specific upregulated processes were identified using cluster-specific upregulated genes. The top 5 up- and downregulated processes were included in bar plot. All terms included met a significance threshold of *P* value of less than 0.05 after correction for FDR < 5%. For details, please see GO Enrichment Analysis in Supplemental Methods.

### GSEA across patient clusters.

GSEA was performed using the R package clusterProfiler (74) (version 3.18.1). Comparison was made to the Hallmark (H), curated (C2), TF target (C3), and GO set (C5) libraries (75). Only terms with a *P* value adjusted for FDR < 5% were included in the analysis.

### TF and biological process network assembly.

GO enrichment analysis was performed as described above. The R package Enrichr (76) (version 3.0) was used for TF target analysis using patient cluster–specific upregulated transcripts. TFs and biological processes were then used as nodes for network assembly with the Linkcomm package in R (77) (version 1.0-14). Linkage between biological processes and TFs was accomplished via shared gene membership between network nodes. Edges, therefore, represent shared gene membership between biological process or TF enrichment terms. For details and code for implementation of this process, please see TF2GO in Supplemental Materials.

### Supervised modeling of k-means patient clusters.

Previous work demonstrated a relationship between the categorical output of k-means clustering and PCA (22). We performed PCA using the genes used for initial k-means clustering with the R package FactoMineR (78) (version 2.0). In an iterative process, gene lists were first generated using a set number of genes from each eigenvector. These gene lists were then used for sparse-partial least square discriminant analysis training on the original SARP data set using the R package “mixOmics” (79) (version 6.10.8). Model performance was evaluated using plotting of balance error rate (BER) versus number of genes required for clustering (Supplemental Figure 6). Optimum parameters were identified using the “elbow” method on graphically plotted data, whereby the inflection point of the LOESS regression line was identified and the solution yielding the lowest BER with the fewest genes was accepted for further study. For code and further details, please see Supplemental Methods Supervised Participant Classification. Following training, this model was used for IMSA cohort participant classification.

### Modeling determinants of FEV_1_.

Genes included in the selected model for supervised classification were used in modeling FEV_1_ of participants from the SARP and IMSA cohorts using EN-regularized regression analysis from the R package “glmnet” (80) (version 3.0-2) with 154-fold or 65-fold model cross validation, respectively. Model prediction was cross-validated using leave-one-out to estimate FEV_1_ based on an individual’s cell count. Model performance was evaluated via comparison of actual measured FEV_1_% predicted and values derived from EN modeling by Spearman’s rank correlation.

### Immunostaining of tissue slides.

Slides were cleared using Histoclear and rehydrated in stepwise alcohol. Following washing, slides were boiled in citrate antigen retrieval buffer for 20 minutes. After permeabilization with 0.5% triton X-100 in TBS with 2% BSA followed by washing in 1% BSA, slides were incubated with primary antibody at 4°C overnight. Secondary labeling was performed with species-appropriate fluorescent conjugate for 1 hour at room temperature the next day. DAPI was used to label nuclei, and slides were mounted in anti-fade media. Imaging was performed on a Leica confocal microscope. Unstained, primary antibody–only and secondary antibody–only slides were prepared from each patient for comparison. Quantification of fluorescent staining was accomplished using the ImageJ software (version 1.51w). Anti–IL-18R1 antibody was obtained from Sigma-Aldrich (HPA007615; anti–IL-18R1 antibody produced in rabbit). Anti-phJNK antibody was obtained from Cell Signaling Technology (phospho-SAPK/JNK [Thr183/Tyr185], catalog G9; mouse mAb, catalog 9255). Anti–ph–c-Jun antibody was obtained from Cell Signaling Technology (ph–c-Jun [Ser73], catalog 9164).

### Immunostaining of Cytospin preparation.

Endobronchial brushings were washed, and cells were fixed to slides using the Cytospin centrifuge (Thermo Fisher Scientific). Staining was performed as described above. Anti-p65 was obtained from Santa Cruz Biotechnology Inc. (sc-8008). Images were obtained on a Leica confocal microscope. Quantification of mean fluorescence intensity was accomplished using the ImageJ software (version 1.51w). Calculation of IL-18R1^+^, p65 nuclear translocated, or ph–c-Jun nuclear translocated cells was done by reader blinded to patient cluster.

### Statistics.

All statistical analysis was performed using the R computing environment (81) (Version 3.6.1) unless otherwise noted. Statistical testing and methodology are described or in figure legends when appropriate.

### Study approval.

All subjects provided informed consent in accordance with an IRB protocol approved by the University of Pittsburgh. Lung tissue was obtained with consent from patients undergoing clinically indicated VATS biopsy during evaluation of their disease.

## Author contributions

Conceptualization was contributed by SEW and MJC. Methodology was contributed by MJC, SEW, XZ, QW, and AR. Data analysis was contributed by MJC, SEW, AR, HETB, XZ, and QW. Writing was contributed by MJC, SEW, and AR. Supervision was contributed by SEW, AR, and MJC. Funding acquisition was contributed by SEW and MC. Data development was contributed by NK and HETB.

## Supplementary Material

Supplemental data

Supplemental data 1

## Figures and Tables

**Table 1:**
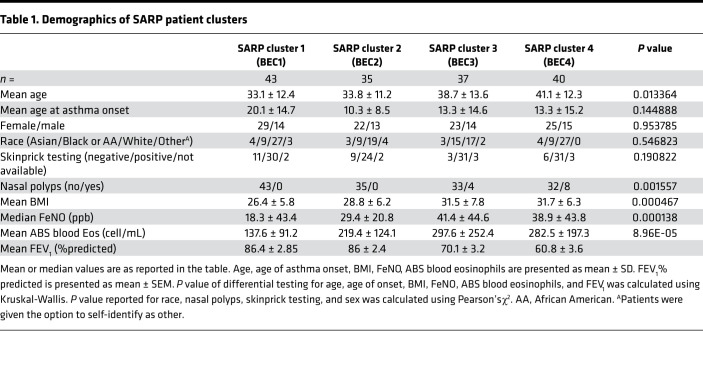
Demographics of SARP patient clusters

## Version 1 09/30/2021

In-Press Preview

## Version 2 11/08/2021

Electronic publication
