# Supplementary material for: Machine learning implicates the IL-18 signaling axis in severe asthma
Source: JCI Insight. 2021 Nov 8;6(21):e149945. doi: 10.1172/jci.insight.149945 (PMC8663569; doi:10.1172/jci.insight.149945)
Supplement: Supplemental data [file jciinsight-6-149945-s172.pdf]

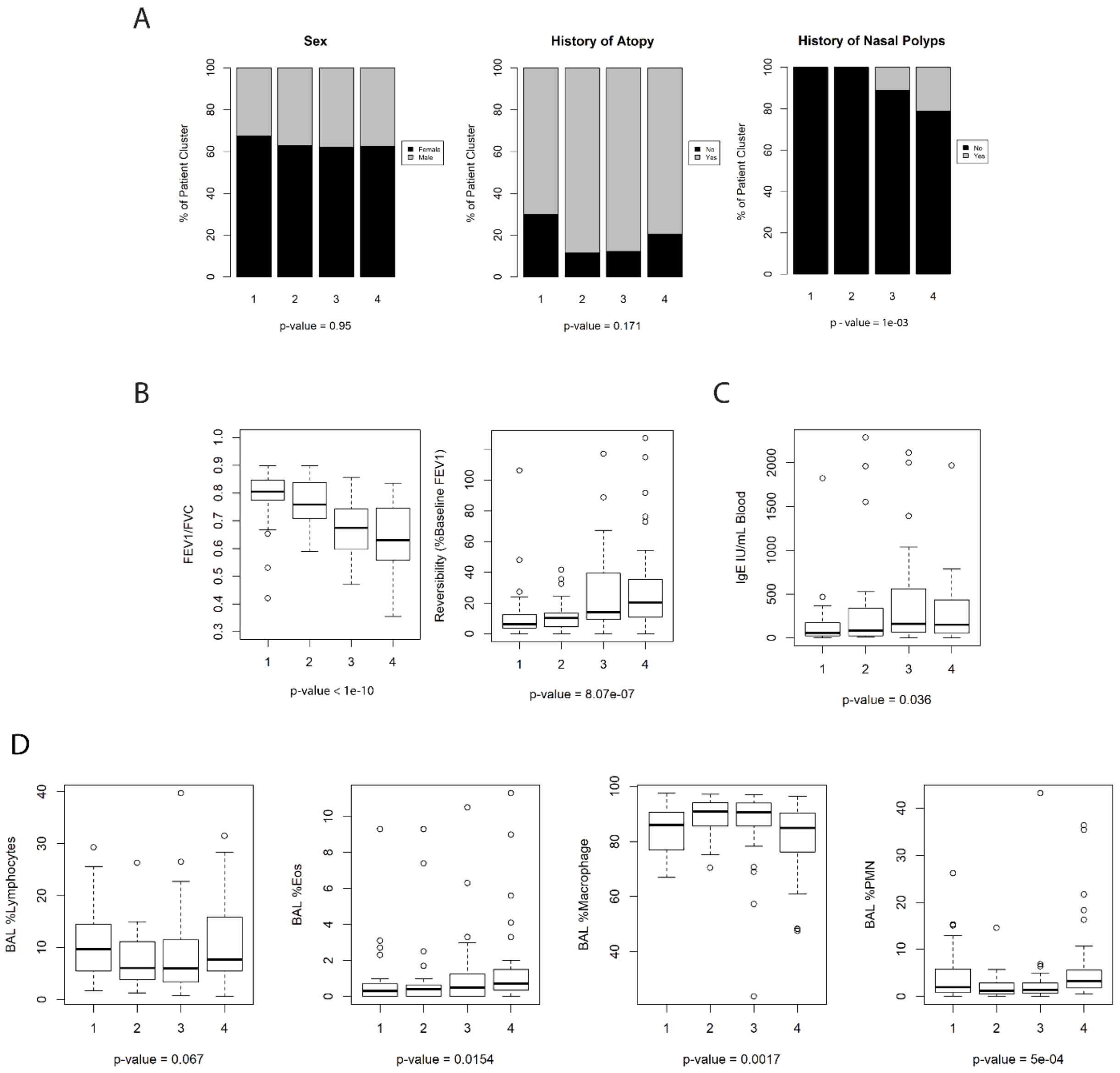

Supplemental Figure S1

(A) Breakdown of participant sex, history of atopy or history of nasal polyps across patient clusters presented as stacked bar chart with p-value from Pearson's chi square testing. (B) Ratio of FEV1 to FVC or reversibility in percentage of FEV1 across patient clusters groups with p-value calculated using Kruskal-Wallis testing. Bars represent median values with bounds of boxes representing interquartile range (IQR) and whiskers representing 1.5x the upper or lower IQR. (C) Blood IgE concentration across patient clusters with p-value calculated using Kruskal-Wallis testing. (D) Percentage of bronchoalveolar lavage (BAL) cell lymphocytes, eosinophils, macrophages and neutrophils (PMNs) across patient cluster with p-value calculated using Kruskal-Wallis testing.

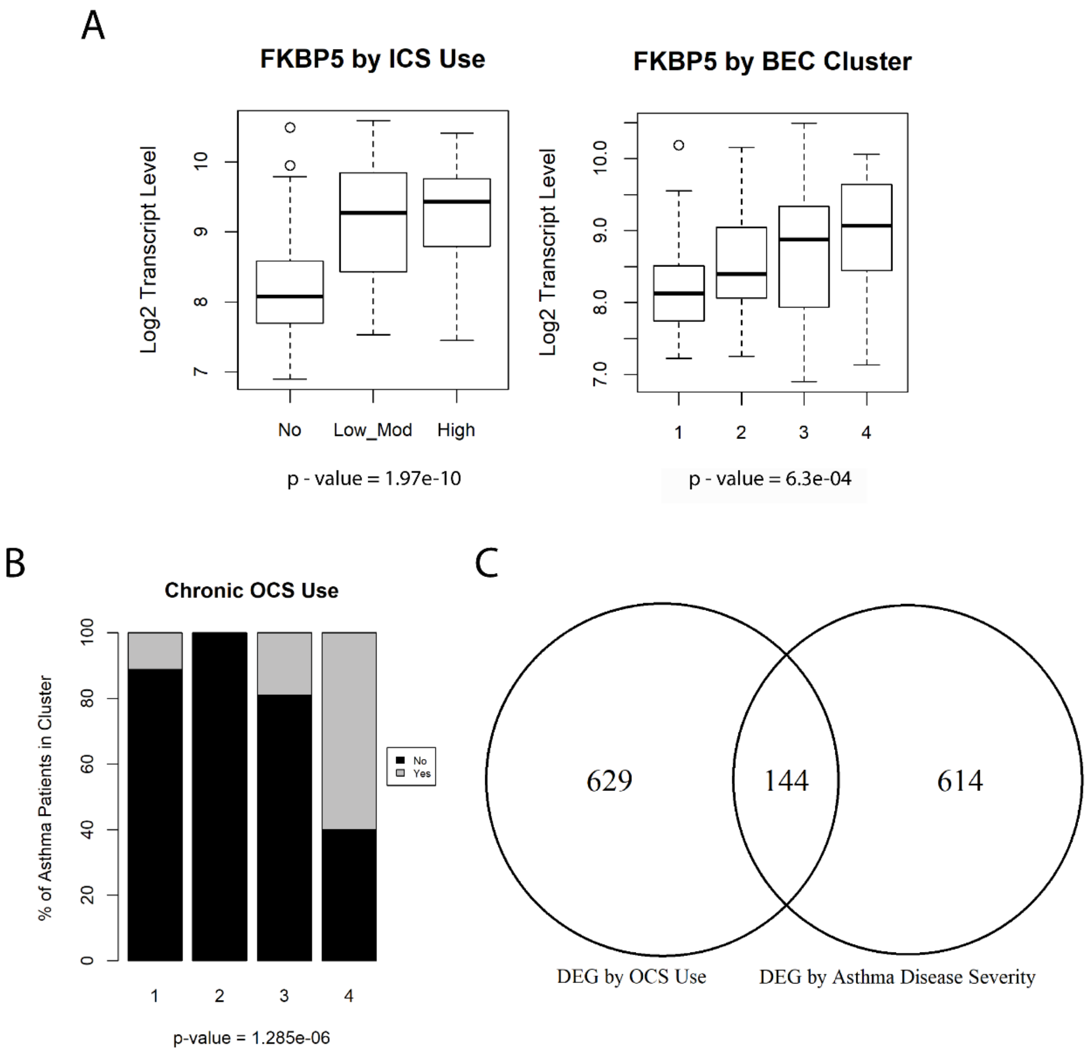

Supplemental Figure S2

(A) FKBP5 transcript level by inhaled corticosteroid use or SARP patient cluster with p-value calculated using Kruskal-Wallis testing. Bars represent median values with bounds of boxes representing interquartile range (IQR) and whiskers representing 1.5x the upper or lower IQR. (B) Breakdown of chronic oral corticosteroid (OCS) use across patient clusters presented as stacked bar chart with p-value from Pearson's chi square testing. (C) Venn diagram demonstrating overlap of genes that were differentially expressed between clinical disease severity classes and healthy controls and genes that were differentially expressed between OCS users and non-users after controlling for sex, inhaled corticosteroid use and clinical disease severity.

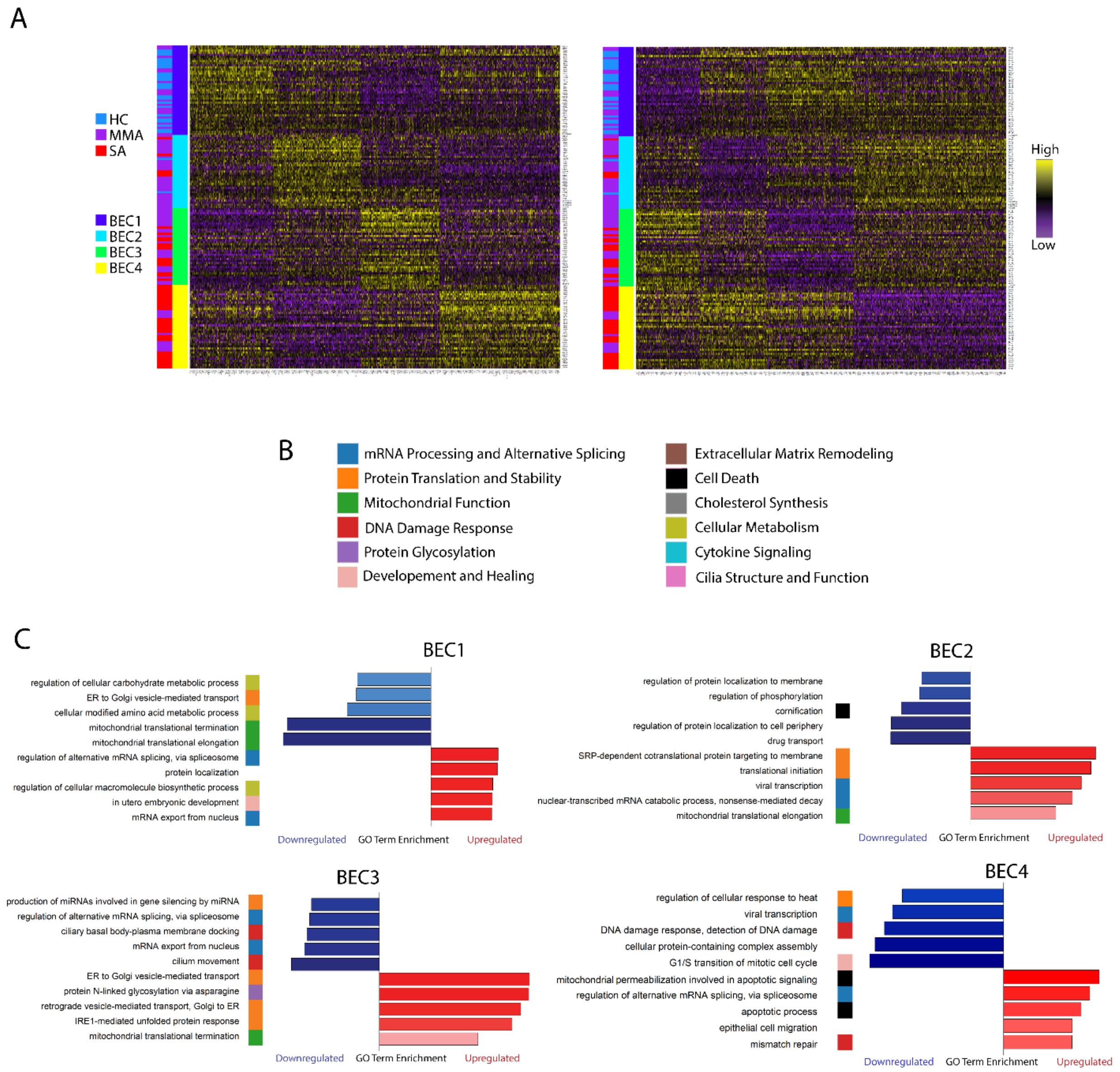

Supplemental Figure S3

(A) Heatmap of genes up- or down-regulated in each SARP patient cluster after controlling for sex and corticosteroid use. Row sidebar indicates patient cluster or clinical disease severity. (B) Key of summary terms represented in Figure 2 as well as Supplemental Figures S3 and S4. (C) Barplot of -log10 p-values for Gene Ontology term enrichment from upregulated (shown in red) or downregulated (shown in blue) transcripts from differential expression analysis. Color coding next to specific terms is used to indicate relationship to summary terms across figures. All specific terms met a cutoff p-value of less than 0.01 after correction for a false discovery rate of less than 5%.

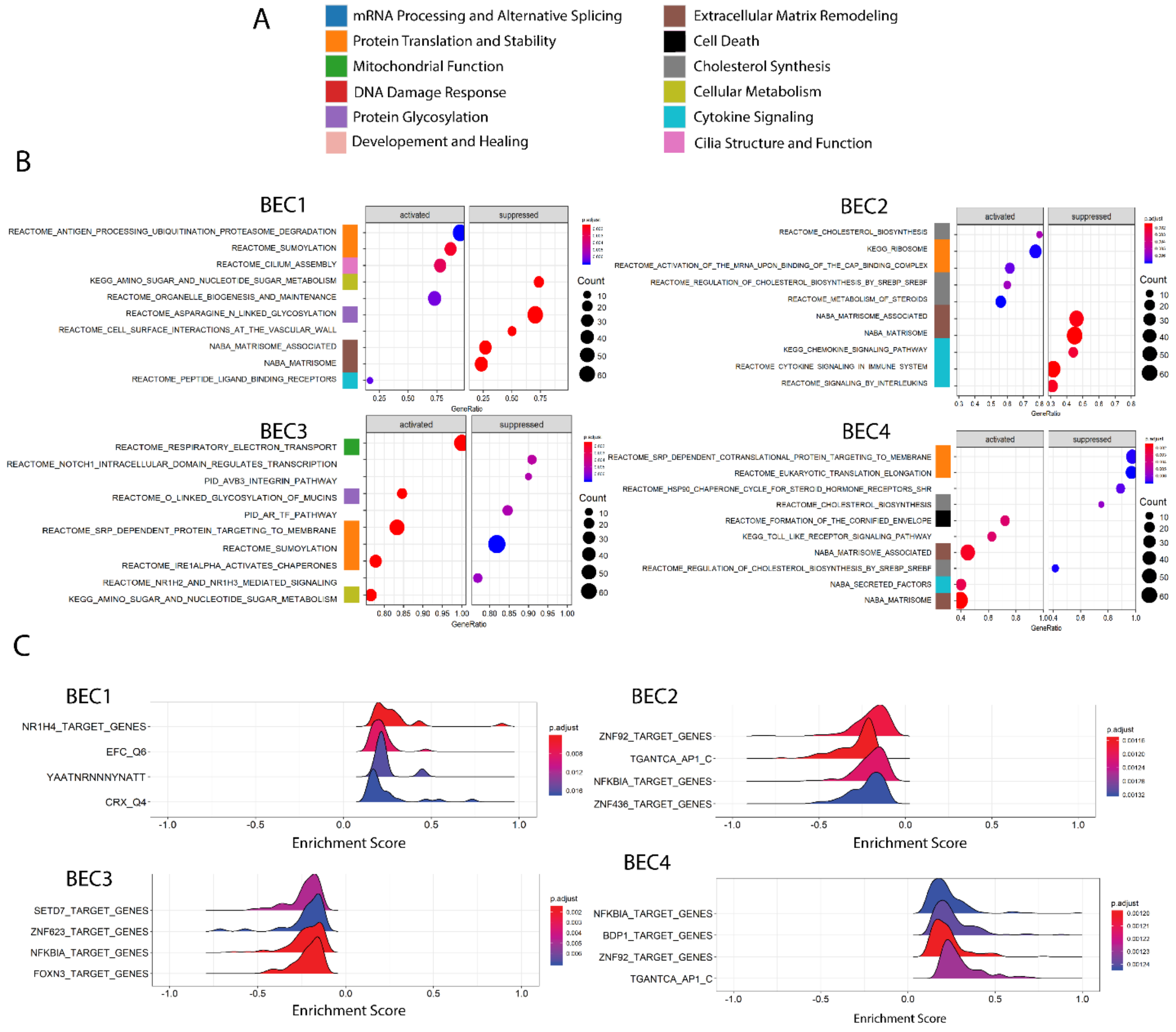

Supplemental Figure S4

(A) Key of summary terms represented in Figure 2 as well as Supplemental Figures S3 and S4. (B) Gene set enrichment analysis (GSEA) of each patient cluster versus the remaining participants with activated and suppressed gene sets from the Hallmark, C2 and C5 libraries as indicated in the plot area. Color of circle in plot area indicated p-value according to adjacent scale. Size of circle corresponds to number of genes in set. Distance on x-axis indicates ratio of genes that are upregulated (activated plot area) or downregulated (suppressed plot area) to total genes in the geneset. (C) GSEA of transcription factor target enrichment in SARP patient clusters versus remaining participants. Distance from origin on x-axis indicates magnitude of enrichment (positive values) or depletion (negative values) of targets in tested patient cluster.

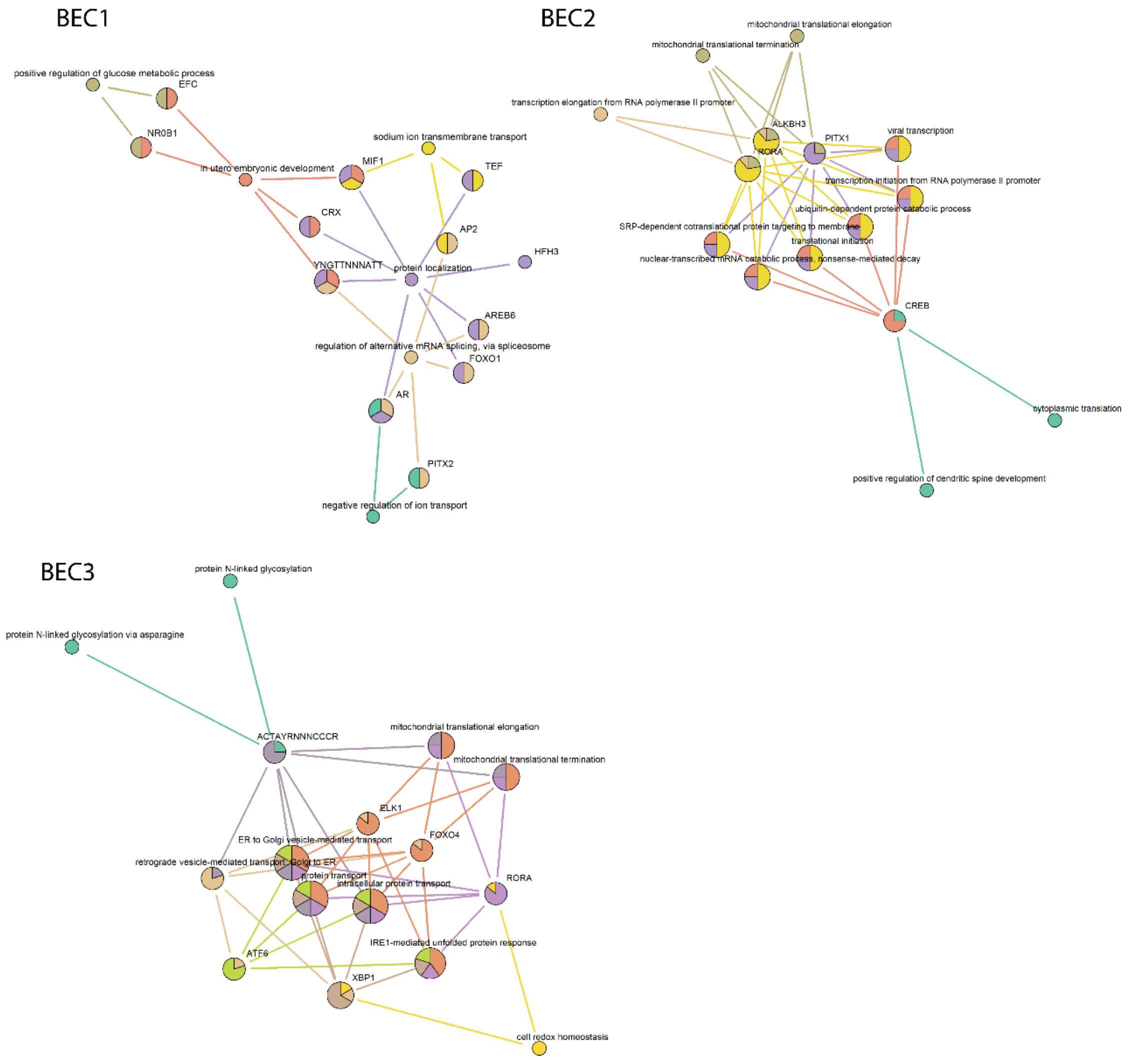

Supplemental Figure S5

Network connectivity graphs between biological processes (BP) and transcription factor target (TFT) sets enriched in indicated SARP patient clusters where nodes represent individual BPs or TFTs and edges represent significant overlap in enriched genes as defined by p-value of hypergeometric overlap $< 10^{-8}$. Edges are colored according to parent BP or TFT and nodes are colored to demonstrate relative enrichment of connected gene sets.

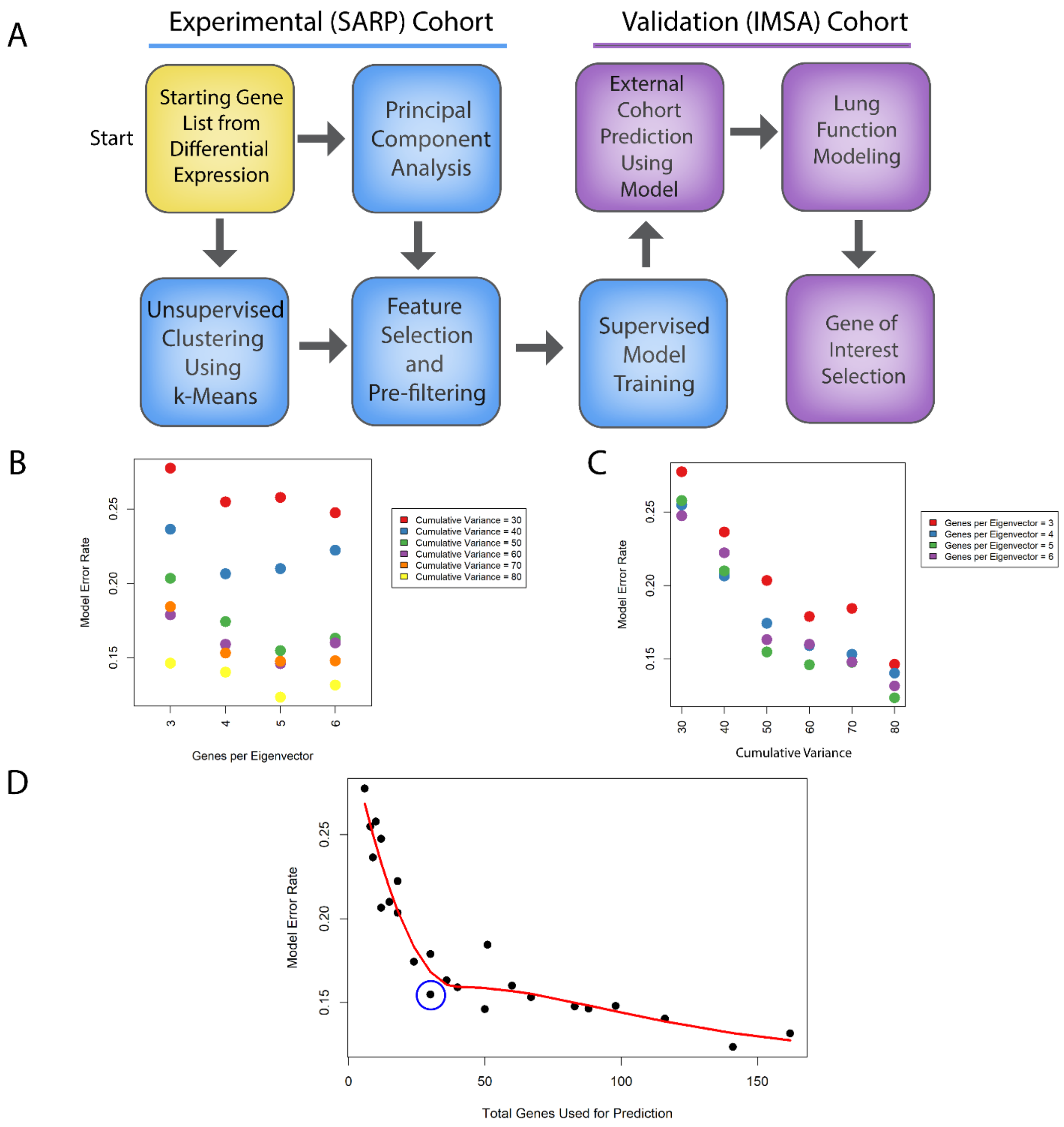

Supplemental Figure 6

(A) Schematic for supervised learning model of patient classification in external (IMSA) cohort using training based on transcriptional profile from SARP. (B) Plot of supervised model error rate vs genes used per eigenvector for differing representations of cumulative variance from the initial data set. Points are colored by cumulative variance calculation from PCA during gene list selection phase. (C) Plot of supervised model error rate vs cumulative variance represented by eigenvector selection. Points are colored by genes used per eigenvector. (D) Plot of supervised model error rate during training on SARP data set versus total number of genes used in the model. Features (genes) were selected based on representation of total variance from PCA prior to use in model. Red line indicates LOESS regression model. Blue circle indicates optimal solution based on the “elbow” method.

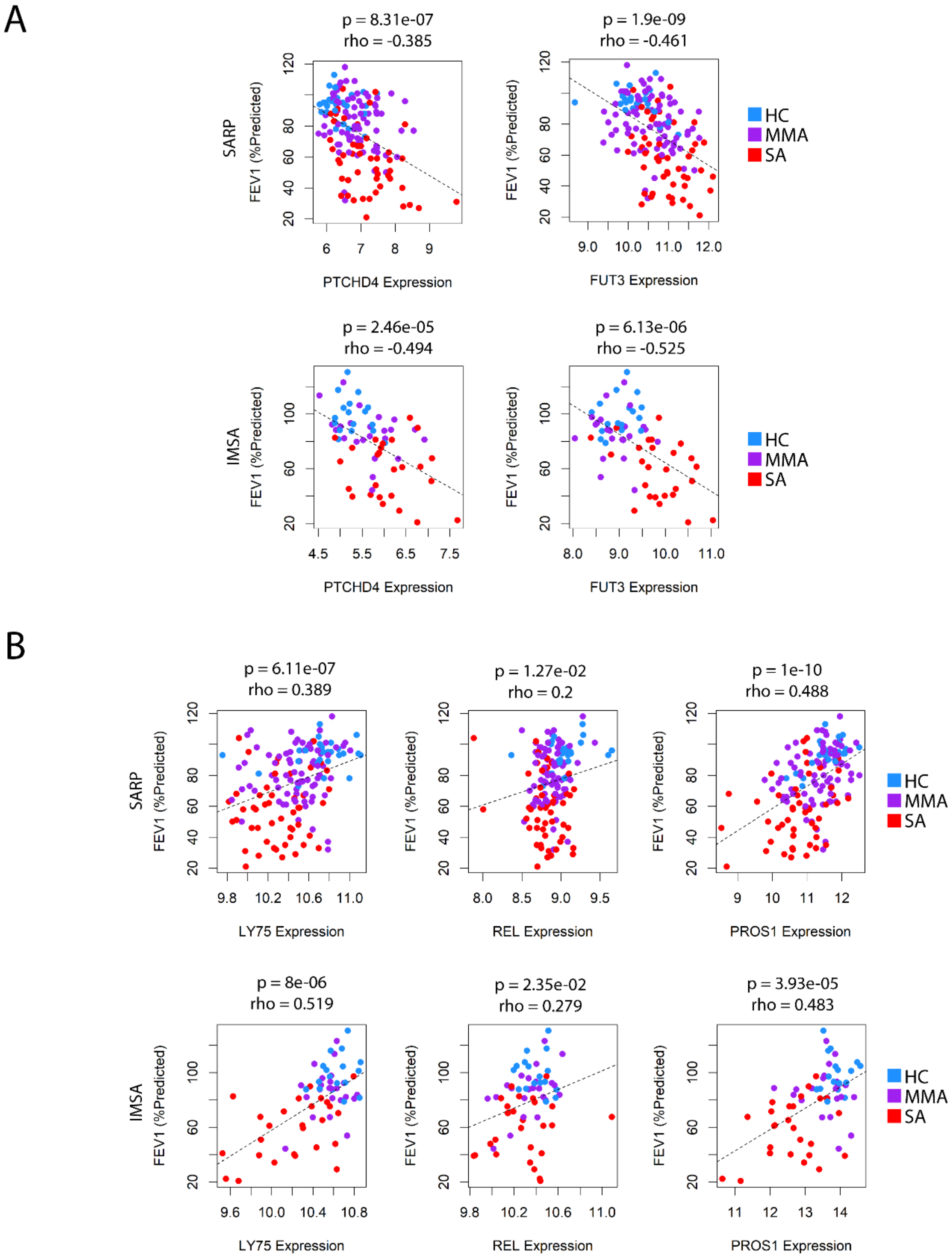

Supplemental Figure 7

(A) Plot of FEV1% Predicted vs. genes identified by EN modeling as having negative relationship to lung function in the SARP and IMSA cohorts. Hashed line represents a linear regression model comparing the two. Spearman's rho and p-value are indicated in plot area. Data points are colored according to clinical disease severity. (C) Plot of FEV1% Predicted vs. genes identified by EN modeling as having positive relationship to lung function in the SARP and IMSA cohorts.
